# Comparison of selective intra-arterial to standard intravenous administration in percutaneous electrochemotherapy (pECT) for liver tumors

**DOI:** 10.2478/raon-2025-0017

**Published:** 2025-02-27

**Authors:** Tim Wilke, Erschad Hussain, Hannah Spallek, Francesca de Terlizzi, Lluis M Mir, Peter Bischoff, Andreas Schäfer, Elke Bartmuß, Matteo Cadossi, Alessandro Zanasi, Michael Pinkawa, Attila Kovács

**Affiliations:** 1 Departement of Gastroenterology, Sinzig Medical Care Center, Linz/Rhein, Germany; 2 Campus Lübeck, University Schleswig-Holstein, Lübeck, Germany; 3 Clinic for Gynaecology and Obstetrics, University Hospital Mannheim, Mannheim, Germany.; 4 IGEA Clinical Biophysics, Laboratory Carpi, Modena, Italy; 5 METSY UMR 9018, Université Paris-Saclay, CNRS, Gustave Roussy, Villejuif, France; 6 Clinic for Diagnostic and Interventional Radiology and Neuroradiology, WEGE Klinik, Bonn, Germany; 7 Clinic for Radiotherapy and Radiation Oncology, WEGE Klinik, Bonn, Germany

**Keywords:** electrochemotherapy, percutaneous ECT, liver metastasis, liver cancer, intra-arterial administration, bleo-mycin

## Abstract

**Background:**

Electrochemotherapy (ECT) is a local nonsurgical effective tumor treatment in the hand of the clinician for the treatment of patients with liver tumors or metastases. The study aimed to test the technical feasibility and safety of intra-arterial (i.a.) bleomycin administration compared to the established intravenous (i.v.) administration in percutaneous electrochemotherapy (pECT). Furthermore, the equivalence hypothesis was tested between the 2 modalities in terms of local short-term response and progression-free survival.

**Patients and methods:**

Forty-four patients have been recruited and treated by pECT for hepatocellular carcinoma, cholangiocarcinoma and liver metastatic lesions from cancers of different origin: 18 were treated with standard i.v., 26 with bleomycin i.a. administration.

**Results:**

The 2 groups were similar for anagraphic and anamnestic data, as well as for most relevant disease specific characteristics. Technical success of the treatment was obtained in 95% and 100% of patients in i.v. and i.a. groups respectively. Short-term local response was similar in the 2 groups with a slightly higher complete remission (CR) rate in the i.a. group. There were 61.9% CR, 23.8% partial remission (PR), 4.8% stable disease (SD) in the i.v. group, and 80.6%, CR 12.9% PR, 3.2% PD (p = 0.3454). One-year progression free survival was 60% (C.I. 33%–88%) in the i.v. group and 67% (C.I. 42%–91%) in the i.a. group (p = 0.5849).

**Conclusions:**

The results of this study confirmed the safety and feasibility of super-selective i.a. bleomycin administration. Analysis of local response and progression free survival confirmed the equivalence hypothesis of the new modality compared to standard i.v. administration in the treatment of primary and secondary liver malignancies by pECT.

## Introduction

Electrochemotherapy (ECT) is a local treatment that utilizes electric pulses application to deliver poorly permeant drugs, such as bleomycin and cisplatin, to cells by increasing permeability of the cell membrane.^1,2^ Over the past 20 years, ECT proved to be effective in the treatment of cutaneous, subcutaneous, mucosal, or deep-seated tumors of various histologies and in different body sites.^3–5^

Since the results of the international, multicentre clinical study European Standard Operating Procedures for Electrochemotherapy (ESOPE) were published and were used to establish the Standard Operating Procedures for ECT on cutaneous tumors with the Cliniporator™ device (IGEA S.p.A., Carpi, Italy), a notable number of preclinical and clinical studies have been conducted on ECT by an European working-group of clinicians to confirm its safety and effectiveness on cutaneous tumors, such as malignant melanoma^6,7^, breast cancer^8,9^, basal cell carcinoma^10^, squamous cell carcinoma^11^ and others.^3,12,13^ Based on these experiences, ECT application was extended and had been shown indeed to be feasible, safe, and effective for deep-seated tumors, such as liver, pancreatic and bone tumors and metastases.^14–19^

In particular, in liver malignancies, either primary or secondary, ECT can be used near collagenous structures such as vessels and bile ducts^20^, it is repeatable and also suitable as a local therapy between chemotherapy cycles. Indeed, non-surgical interventional local tumor treatments are currently an additional option for the treatment of cancer patients and The European Society of Medical Oncology (ESMO) included local therapies in the current consensus guidelines on the treatment of metastatic colorectal cancer (mCRC).^21^

Local treatments for the management of liver malignant lesions can be divided into thermal (radiofrequency or microwave ablation and cryoablation) and nonthermal treatments (high precision radiotherapy, brachytherapy, irreversible electroporation and ECT).^14,19^ All these treatments are ablative. Moreover, ECT is a method to vectorize the chemotherapy, being therefore highly selectively against the tumor cells whilst preserving the normal cells.

The choice of the therapy for each single case is determined by the number, size, configuration and location or environment of the target lesion. Contrary to Irreversible Electroporation (one supplementary ablative technique), ECT is also a selective approach for the vectorization of cytotoxic drugs and has gained a role in the armamentarium of local therapies available to the clinicians, enabling the treatment of i) lesions that are too large for thermal ablation, ii) non-radiosensitive tumors or iii) lesions located in the immediate vicinity of radiation- or temperature-vulnerable organs.^14^ Specifically, ECT can be used in the treatment of liver lesions located centrally, close to the capsule or in proximity of the major vessels and bile ducts, which may neither be resectable nor suitable for radiofrequency or microwave ablation. The safety of ECT in the treatment of lesions located near large liver vessels was also proven in animal models.^22,23^ Good tolerance, with few side effects and no relevant pain, nausea or systemic side effects were also observed.^24–26^

Conventional percutaneous ECT (pECT) in the liver is performed with intravenous (i.v.) administration of bleomycin following the Standard Operating Procedures for ECT in cutaneous and subcutaneous lesions.^27,28^

A novel procedure for the administration of bleomycin in the liver has been introduced, based on the already established liver-directed endovascular therapies, hepatic artery infusion (HAI) and transarterial chemoembolization (TACE): the selective delivery of bleomycin intra-arterially to the lesion area to be treated. The vessels supplying the lesion are accessed from the groin via a guiding catheter and a coaxially used microcatheter. The complete volume coverage of the entire lesion is ensured by contrast-enhanced Cone-Beam Computed Tomography (CBCT) scan. In this way bleomycin is directly delivered selectively into the lesion with higher concentration in loco. Since selective and superselective chemotherapy applications are already well established in interventional oncology, the aim of this work is to translate this established approach in the ECT setting. We aimed to test the technical feasibility and safety of this approach and to demonstrate the equivalence of intra-arterial (i.a.) bleomycin administration compared to the established i.v. administration.

In this study, the new method of administration is fully described and named “the intra-arterial pECT method”.

## Patients and methods

### Patients

Forty-four patients have been recruited and treated by ECT in our centre for hepatocellular carcinoma, cholangiocarcinoma and liver metastatic lesions from cancers of different origin: colorectal cancer, breast cancer, ovarian cancer, anal cancer, non-small cell lung cancer (NSCLC), pancreatic cancer, parotid carcinoma, neuroendocrine carcinoma, uterus carcinoma, cancer of unknown primary origin (CUP) and oesophageal carcinoma. Eighteen patients were treated with conventional intravenous administration of bleomycin (i.v. group), whilst twenty-six were treated using intra-arterial administration of the same drug (i.a. group). ECT treatments were performed between June 2018 and June 2024. The study was conducted according to the WMA Declaration of Helsinki – Ethical Principles for Medical Research Involving Human Subjects. All patients signed an informed consent form and agreed to be included in the data collection. The study was approved by the Committee for Medical Ethics of the Institution (*Ethik Kommission der Ärztekammer Nordrhein* Nr 2022314).

### Imaging

Standard pretreatment evaluation of patients was performed including MRI (magnetic resonance imaging) with hepatospecific contrast agent and thorax plus abdomen CT (computed tomography), including also the pelvis, not more than 30 days before treatment. Details on MRI are reported in Spallek *et al*.^14^

### Electrochemotherapy with conventional intravenous bleomycin administration

Software-based treatment planning for the correct positioning of the electrodes was performed on MRI preoperative images. The aim of the treatment planning is to obtain the electric field optimal coverage of the target lesions by including them within the needle geometry. A maximum of 6 electrodes can be activated synchronously by the pulse generator, but various spatial domains can be treated during the same session with the use of more than 6 electrodes. The needle electrodes were percutaneously inserted in and around the lesions following multimodal image guidance and stereotactic navigation, at a minimum/maximum distance of 0.5/3.0 cm one from the other. Needle electrodes have a diameter of 1.2 mm and a 16 to 24 mm length, with an active part of 3 or 4 cm long (IGEA^®^, Carpi, Italy). They are freely positionable and must be inserted in parallel, to ensure the correct delivery of the electric field. The direction of access of the electrodes was determined by the performing surgeon.

Once positioned, the electrodes were supplied by a suitable voltage to deliver sequential electric pulses. The goal is to ensure complete coverage of the clinical target volume with an electric field intensity above 400 V/cm and to maintain the maximum current delivered below 50A. The electric pulses were delivered by the CliniporatorTM device (IGEA^®^, Carpi, Italy).

When administering bleomycin intravenously, the same treatment protocol as defined by the SOP for ECT of cutaneous tumors was adopted^27,28^ with regard to drug administration, dose, and electrical parameters (i.e. pulse duration and number of pulses). After having correctly positioned the electrodes, under general anesthesia, the patients were given bleomycin at a dose of 15.000 IU/m^2^ body surface intravenously in bolus within 30 seconds. Eight minutes after the bleomycin administration the maximal pharmacological peak of bleomycin in the organs is expected and it is possible to start the electroporation process in liver, delivering 8 pulses of 100 μs duration between each pair of electrodes. Pulses delivery should be completed within 40 minutes.

Care was also taken to ensure that the electrical pulses were delivered only in the refractory phase of the heart by synchronizing of the Cliniporator device with the ECG (electrocardiogram) to avoid interferences with the heart rhythm.

### Electrochemotherapy with intra-arterial bleomycin administration

After a local anesthesia, retrograde puncture of the right common femoral artery and insertion of a 4F sheath system were performed. The vessels supplying the tumor were probed super-selectively using a guiding microcatheter (Sidewinder 4F, Terumo^®^) and microcatheter (Persue 2.0 Swan Neck, Merit^®^) in coaxial technique. The complete volume coverage of the entire tumor was assessed in CBCT, simulating the subsequent bleomycin distribution. The microcatheter was then secured in position and the patient was transferred to the interventional CT. In the CT, the target lesion was visualized by a super-selective contrast via the microcatheter (10 cc of pure CT-contrast, followed by 10 cc saline each 1 cc/sec) and this volume data set was used for navigation. The electrodes were positioned under general anesthesia using multimodal image guidance and stereotactic navigation (CAS One, Cascination^®^), with the same precautions adopted for the i.v. approach. A second i.a. contrast after electrode-placement was applied to confirm adequate coverage of the lesion, including safety margins. As soon as the electrodes were correctly positioned, 50% of the total bleomycin volume (total bleomycin dose: 15000 IU/m^2^ body surface) was administered intra-arterially via the microcatheter in a continuous injection. When about 50% of drug was infused, the electric pulses delivery started and during the electric pulses′ delivery, the remaining 50% of the bleomycin continued to be administered intra-arterially. The injection of 100% bleomycin before electric pulses′ delivery was discarded because, due to the first pass effect, there is a risk that a relevant part of the drug has passed through the tumor, making it unavailable for electroporation. After the electric pulses′ delivery, a CT scan was performed to rule out therapy-associated complications. After successful electric pulses delivery, the electrodes, catheter and sheath were removed, and the puncture site was manually compressed followed by the subsequent removal of the electrodes and the application of a sterile plaster dressing. The catheter and sheath material were then removed, followed by fifteen minutes of manual compression of the puncture site until hemostasis was achieved. Then, application of a sterile plaster bandage and a pressure bandage were applied. Further standard procedures were adopted: patient monitoring, laboratory checks and post-interventional imaging according to ward protocol.

### Response to treatment evaluation

Response to treatment was evaluated based on the multiparametric MRI of the liver, including multiplanar ce T1w, transversal T2w fs and transversal DWI scans at 1 to 3 months follow-up. Lesion-based treatment success was assessed using the Modified Response Evaluation Criteria in Solid Tumours (mRECIST) in terms of complete remission (CR), partial remission (PR), stable disease (SD), and progressive disease (PD). Local tumor control was defined as CR, PR or SD according to the RECIST criteria, version 1.1.^29^ Objective response is obtained by the sum of CR and PR.

### Statistical analysis

Continuous variables are reported as the mean and standard deviation, median and range. Categorical variables are expressed as absolute numbers and percentages. Comparisons between the two groups were performed by heteroschedastic 2 tails Student t-test for continuous variables and contingency tables with Chi square calculation for categorical variables. Progression free survival time was calculated in months as the time since ECT session date to last follow-up (in case of no progression) or to the date of progression. Progression free survival analysis was conducted by means of calculation of Kaplan Meier survival curves and logrank test for comparison between groups. A p value lower than 0.05 is considered statistically significant. Statistical analysis was performed with NCSS 9 (NCSS 9 Statistical Software [2013]).

## Results

Patients were enrolled and treated in the period between June 2018 and June 2024 and were followed for a median time of 7 months (range 1–27; mean 7.8 ± 5.9 months). They were divided into two groups based on conventional intravenous or intra-arterial administration of bleomycin; 18 patients were treated with conventional i.v. and 26 with i.a. administration of chemotherapy drug.

Mean age in the two groups was similar (63 ± 11 yrs, median 64 range 41–83 in the i.v. group vs 68 ± 10 yrs, median 69 range 52–93 in the i.a. group, p = 0.2182). The two groups were similar for almost all characteristics reported in Table 1.

**TABLE 1.** Descriptive characteristics of the patients in the 2 groups

PATIENTS	i.v. pECT (N = 18)		i.a. pECT (n = 26)		P value
	N	%	N	%	
**Gender**					
**Males**	8	44%	15	58%	0.5406
**Females**	10	56%	11	42%	
**Diagnosis**					
**Colorectal cancer**	7	39%	10	38%	0.4620
**Breast cancer**	4	22%	4	15%	
**Hepato cellular carcinoma**	2	11%	2	8%	
**Cholangio cellular carcinoma**	0	0%	3	12%	
**Ovarian cancer**	2	11%	0	0%	
**Non-small cell lung cancer (NSCLC)**	1	6%	2	8%	
**Anal cancer**	1	6%	0	0%	
**Pancreatic cancer**	0	0%	1	4%	
**Parotis carcinoma**	0	0%	1	4%	
**Neuroendocrine carcinoma**	0	0%	1	4%	
**Uterus carcinoma**	0	0%	1	4%	
**Esophageal carcinoma**	0	0%	1	4%	
**Cancer of unknown primary origin (CUP)**	1	6%	0	0%	
**Liver metastases**					
Synchronous	8	44%	16	62%	0.5343
Metachronous	8	44%	8	31%	
No	2	11%	2	7%	
**Metastases location other than liver**					
None	9	50%	17	65%	0.3613
Yes	9	50%	9	35%	
**Other metastasis’ location**					
Lung	3	17%	3	12%	0.6025
Bone	1	6%	2	8%	
Kidney	1	6%	1	4%	
Lung + bone + brain	1	6%	0	0%	
Bone + peritoneum	1	6%	0	0%	
Pleural + bone	1	6%	0	0%	
Retroperineal	1	6%	0	0%	
Adrenal gland	0	0%	1	4%	
Lymphnode	0	0%	1	4%	
Bone + lymphnode	0	0%	1	4%	
**Previous treatments**					
Systemic therapy	16	89%	26	100%	0.1617
Liver surgery	4	22%	12	46%	0.1251
Local treatments	11	61%	8	31%	0.0657
**Type of local treatments**					
TACE	8	43%	4	15%	0.1529
TACE+RFA	1	6%	0	0%	
TACE+CP	1	6%	0	0%	
TACE/MWA/CRYOTH	0	0%	2	8%	
CRYOTH	1	6%	0	0%	
IBT	0	0%	2	8%	
**Comorbidities**					
Cardiac diseases	6	33%	7	27%	0.7422
Pulmonary diseases	3	17%	7	27%	0.4889
Liver diseases	9	50%	0	0%	<0.0001
**Number of target lesions per patient**					
1	15	83%	21	81%	1.000
2	3	17%	5	19%	

1 CP = chemoperfusion; CRYOTH = cryotherapy; IBT = interstitial brachytherapy; i.a. = intra-arterial; i.v. = intravenous; MWA = microwave ablation; pECT = percutaneous electrochemotherapy; RFA = radiofrequency ablation; TACE = hepatic artery chemoembolization

In total 21 lesions were treated in the i.v. group, and 31 in the i.a. group. Lesions′ size was significantly larger in the i.v. group, with a mean LA (long axis) size of 5.9 ± 2.5 cm (median 4.6 cm, range 1.5–11.2), with respect to i.a. group, with a mean LA size of 4.4 ± 2.2 (median 4.0 cm, range 1.1–9.7) (p = 0.0300). Volume mean values on the other hand show only a slight not significant difference: 130 ± 137 cm^3^ in the i.v. group vs 61.4 ± 81.4 cm^3^ (p = 0.0501). Characteristics of the target lesions are listed in Table 2. Mean number of electrodes used in the i.v. group is 5.5 ± 1.4 (median 6, range 2–8) and in the i.a. group it is 5.0 ± 1.0 (median 5, range 3–6), values not significantly different (p = 0.1380).

**TABLE 2.** Characteristics of target lesions in the 2 groups

LESIONS	i.v. pECT (N = 21)		i.a. pEC (N = 31)		P value
	N	%	N	%	
**Type**					
Hypervascular	2	9.5%	5	16.1%	0.7664
Intermediate	14	66.7%	20	64.5%	
Hypovascular	5	23.8%	6	19.4%	
Challenging location*					
Yes	19	90.5%	21	67.7%	0.0927
No	2	9.5%	10	32.3%	
**Vessels or bile ducts surrounding the metastases**					
Distant (> 10 mm)	4	19.0%	8	25.8%	0.5389
Close (1 mm to 10 mm)	6	28.6%	5	16.1%	
Adjacent (< 1 mm)	11	52.4%	18	58.1%	
**Previous local treatments on the lesion**					
Yes	7	33.3%	8	25.8%	0.7559
No	14	66.7%	23	74.2%	
**Type of local treatments on the lesion**					
TACE (transarterial chemoembolization)	6	28.6%	4	12.9%	0.1474
CP (chemoperfusion)	1	4.8%	0	0.0%	
IBT (interstitial brachytherapy)	0	0.0%	2	6.5%	
TACE/MWA/CRYOTH	0	0.0%	2	6.5%	
**Technical success**					
Yes	20	95.2%	31	100.0%	0.2199
No	1	4.8%	0	0.0%	

1 * Challenging location represented in liver were liver dome, vicinity of portal vein main trunk, vicinity of main bile duct

1 CP = chemoperfusion; CRYOTH = cryotherapy; IBT = interstitial brachytherapy; i.a. = intra-arterial; i.v. = intravenous; MWA = microwave ablation; pECT = percutaneous electrochemotherapy; TACE = hepatic artery chemoembolization

### Safety/toxicity

Side effects observed in both groups were only mild or moderate, such as mild pain at the treated site, C-reactive protein (CRP) elevation and leucocytosis, or haemoglobin drop. All side effects were successfully treated with appropriate medical treatments and disappeared within 10 days from occurrence. No differences were observed in the two groups with respect to onset of side effects.

### Response to treatment

**FIGURE 1.**
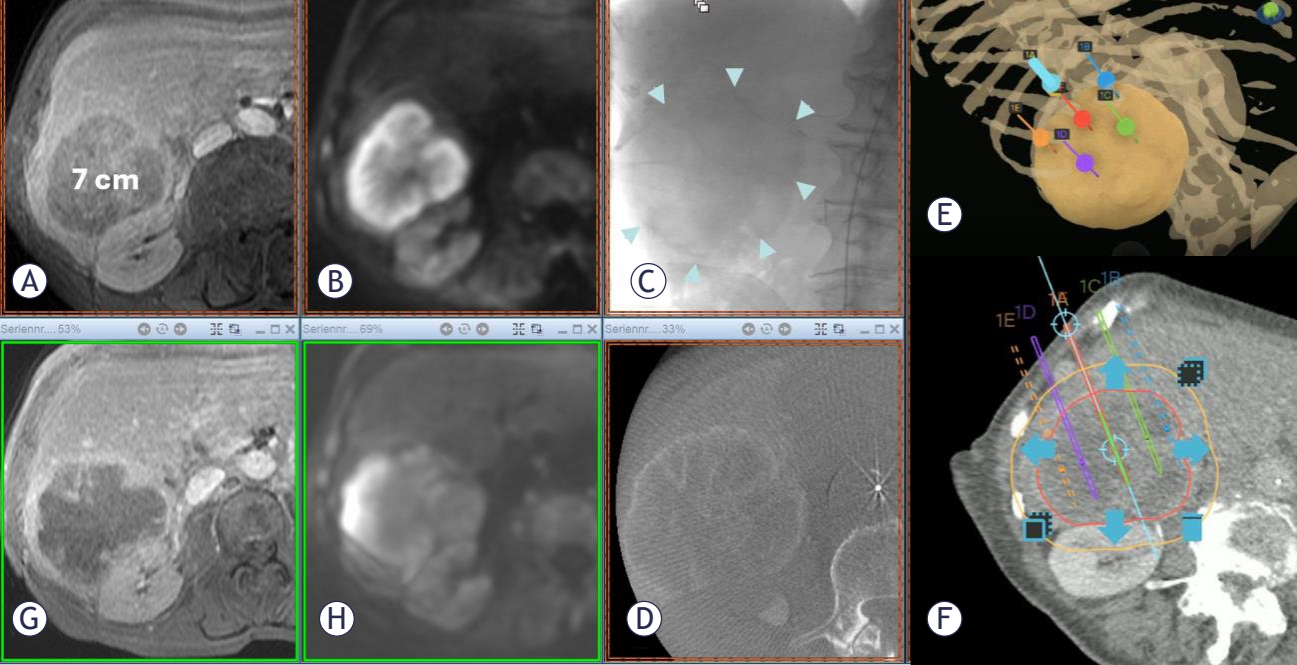
**(A)** Transverse contrast-enhanced T1w MR image of a pancreatic carcinoma metastasis with a maximum diameter of 7 cm in segments V/VI and VII, which no longer responds to systemic chemotherapy and is growing rapidly and progressively. **(B)** Corresponding slice to A in diffusion imaging. **(C)** Complete contrast of the metastasis in 2D angiography. **(D)** Documentation of complete contrast coverage of the metastasis in cone beam CT. **(E)**. Stereotactic navigation (CAS-One^®^ IR) of the electrodes for electroporation. **(F)**. Parallel planning of the electrodes in the tumor to achieve homogeneous electroporation. **(G)**. The transverse contrast-enhanced T1w MR image of the metastasis one month after the electrochemotherapy (ECT) procedure demonstrates the complete loss of perfusion in the entire metastasis. **(H)** Corresponding slice to G in diffusion imaging.

Response to pECT treatment was evaluated for each target lesion within the first 3 months of follow-up and the result for each single group is reported in Table 3. Despite a higher CR rate in the i.a. group, no statistically significant difference has been observed between the groups. Objective response (OR) rate was 85.7% in the i.v. group and 93.5% in the i.a. group (Figure 1).

**TABLE 3.** Response to percutaneous electrochemotherapy (pECT) treatment in the 2 groups

RESPONSE	i.v. pECT (N = 21)		i.a. pECT (N = 31)		P value	P value
	N	%	N	%	Overall distribution	CR *vs*. non CR
**CR**	13	61.9%	25	80.6%	0.3454	0.1349
**PR**	5	23.8%	4	12.9%		
**SD**	1	4.8%	0	0.0%		
**PD**	0	0.0%	1	3.2%		
**Lost to follow up**	2	9.5%	1	3.2%		

1 CR = complete remission; i.a. = intra-arterial; i.v. = intravenous; pECT = percutaneous electrochemotherapy; PR = partial remission; SD = stable disease

**FIGURE 2.**
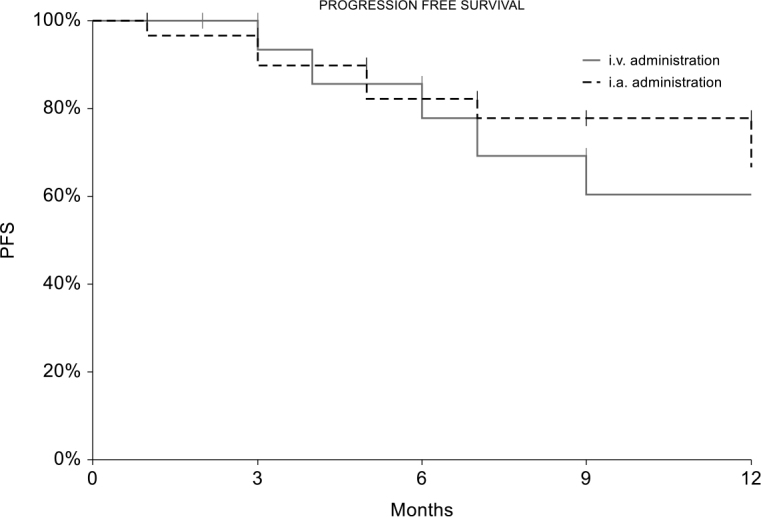
Progression free survival in the 2 groups during the 18-month follow-up period.

Progression free survival is shown in Figure 2. No significant difference has been observed between groups (p = 0.5849). One-year progression free survival is 60% (C.I. 33%–88%) in the i.v. group and 67% (C.I. 42%–91%) in the i.a. group.

## Discussion

This study assesses the feasibility of intra-arterial administration of bleomycin for pECT of liver tumors. Since selective and super-selective chemotherapy applications are already well established in interventional oncology, such as in TACE, HAI and IHP (isolated hepatic perfusion)^30,31^, the rational was to combine this established approach with pECT. Specifically, the goal of intra-arterial therapies is to inflict lethal insult to tumors by selectively delivering anticancer treatment to its arterial supply. Regional delivery of a drug leads to its increased local concentration; this holds true for drugs demonstrating first-order kinetics (constant clearance) despite the higher dose, potentially leading to an increased response. Moreover, regional drug delivery leads to decreased systemic exposure of that drug, potentially reducing side effects and toxicity.^32^ Even in pECT, the administration of the chemotherapy drug only in the tumor-bearing area to be treated, allows a higher concentration of the drug in loco, enabling a first pass effect, with less systemic bleomycin circulating in the body, i.e. decreased risk of side effects and lung toxicity, whilst off-target bleomycin already bearing low toxicity in non electroporated liver parenchyma.

The intra-arterial application of bleomycin in the liver proved to be safe and effective in the treatment of liver haemangiomas, with doses up to 45.000 IU per session^33^, as well as in TACE doxorubicin resistant hepatocellular carcinomas.^34^ Sporadic reports about the intra-arterial application of bleomycin in ECT of other tumor entities can also be found in the literature.^35,36^ To the best of our knowledge, this is the first study showing that the intra-arterial administration of bleomycin can be applied to pECT of liver tumors.

Intra-arterial catheter-assisted pECT also allows real-time liver tumor visualization before, during, and after placement of the probes, which may help in: improving tumor conspicuity; guiding needle advancement; verifying probe position relative to the tumor and surrounding structures; evaluation of procedure-related complications and technical success directly after the procedure, all with a significantly reduced contrast dose comparable to venous contrast and therefore reduced risk of compromising renal function.^37–39^

In this cohort study, intra-arterial administration of bleomycin is proposed as a new method of drug delivery in pECT. Here we compared this new method with the standard intravenous systemic administration of bleomycin in patients undergoing pECT for liver lesions of various histological origins. The 2 groups were similar for age, gender, histology distribution, type of metastasis (synchronous, metachronous), metastasis location, previous treatments, and comorbidities, except for surrounding liver disease. A significant difference in LA size of the lesion is observed (p = 0.0300), even if when calculated the entire volume of the lesion, the difference is only marginally significant (p = 0.0501).

The analysis of complications and side effects reveals that the conventional intravenous and the new intra-arterial methods bring the same risk of complications. Percutaneous ECT is well tolerated by the patients^14,40^, no serious adverse events have been observed during procedures and during follow-up; side effects appear to be limited in number and intensity, and when occurred they were successfully treated with appropriate medical treatments and disappeared within 10 days from occurrence.

Short term results indicate a substantial equivalence between i.v. and i.a. administration modality, as non-significant differences were observed in the outcome at 1 to 3 months of follow-up. Complete response rate was 61.9%, partial response 23.8%, stable disease 4.8% and progression 9.5% in the i.v. group. Interestingly, a slightly higher CR rate was observed in the i.a. group, 80.6% with a lower PR rate, 12.9%; this non-significant difference could be ascribed to the relatively smaller target lesions treated with i.a. procedure of bleomycin administration, with a volume of 61.4 ± 81.4 cm^3^ with a marginal significance in comparison with the i.v. group. In any case the objective response rate in the 2 groups is 85.7% in the i.v. group and 93.5% in the i.a. group. These data are similar to previous experiences on ECT in the liver: Edhemovic *et al*.^25^ conducted a study on intraoperative ECT of colorectal liver metastases and obtained a response rate per patient of 75% (63% CR and 12% PR). In a similar study carried out on intraoperative ECT in hepatocellular carcinoma, Djokic *et al*.^41^ obtained a response rate per patient of 95.8% (79.2% CR and 16.6% PR). The complete response rate at 3–6 months was 80% per patient and 88% per treated lesion, with a median size of the treated lesions of 24 mm (range 8–41 mm), slightly smaller than those treated in our study. Considering the studies on ECT of liver performed with percutaneous insertion of the electrodes, the experience of Tarantino *et al*.^42,43^ on cholangiocellular carcinoma at hepatic hilum and on portal vein tumor thrombosis at hepatic hilum in patients with hepatocellular carcinoma in cirrhosis is also relevant. Even if few patients were included, they could demonstrate for the first time the feasibility and efficacy of the percutaneous procedure in the liver with intra-arterial administration of bleomycin. No important side effects and a complete response was obtained in 3 out of 5 patients and maintained for at least 18 months. Further authors reported their experiences in pECT of liver tumors^24,44^ and more recently Iezzi *et al*. proved the feasibility of performing pECT under analgo-sedation, obtaining 100% OR in 5 patients with liver lesions, reporting neither major nor minor complications.^45^

In our study, the follow-up time ranges from 1 to 30 months, with a median value of 7 months, thus it is not possible to provide a very long time progression free survival evidence, nonetheless the data available show that within the first 12 months of follow-up the progression free survival is similar between the i.v. and i.a. groups (p = 0.5849) and this result confirms the hypothesis of equivalence in the local tumor control of i.a. procedure with respect to i.v. pECT standard procedure. This result is very important because it is complemented by several theoretical advantages of i.a. pECT, such as: targeted drug application only in the tumorbearing area to be treated, higher concentration of bleomycin in loco using the first pass effect, less systemic drug circulating in the body and less side effects, less lung toxicity, better timing of the EP as local infusion is quicker than systemic.

By proving the equivalence of i.a. and i.v. administration, there is also the potential to reduce the bleomycin dose in the future due to the higher on-site concentration with i.a. administration; this might be the aim of further research.

The present study has some limitations: the relatively small number of patients is mainly due to the feasibility aim of the study, and the short follow-up after treatment, even if feasibility and short-term efficacy have been clearly obtained. With a perspective of collecting more data on patients treated with i.a. pECT in the liver, it will be possible to better define the optimal indications for this new procedure with respect to conventional i.v. pECT, even if some indications can already be suggested: based on our experience, for example, for hypovascularized tumors, which seem to respond less well to i.v. ECT than hypervascularised tumors.

## Conclusions

This study demonstrates the equivalence of super-selective i.a. bleomycin administration compared to standard i.v. administration in the context of percutaneous electrochemotherapy for primary and secondary liver malignancies and thus opens perspectives for reducing the bleomycin dose and the associated toxicity.
